# Revalidation of the ATTRACTION-4 study in a real-world setting: a multicenter, retrospective propensity score matching study in China

**DOI:** 10.3389/fimmu.2023.1264929

**Published:** 2023-09-15

**Authors:** Yuhong Dai, Yongqing Liu, Zhimin Gong, Lilin He, Lei Wang, Wenjie Yang, Ping Qiu, Fangyuan Zhang, Xianglin Yuan, Henghui Cheng, Hong Qiu

**Affiliations:** ^1^ Department of Oncology, Tongji Hospital Affiliated to Tongji Medical College of Huazhong University of Science & Technology, Wuhan, Hubei, China; ^2^ Department of Oncology, Xiangyang Central Hospital, Affiliated Hospital of Hubei University of Arts and Science. Institute of Oncology, Hubei University of Arts and Science, Xiangyang, Hubei, China; ^3^ Department of Oncology, The First People’s Hospital of Tianmen, Tianmen, Hubei, China; ^4^ Department of Oncology, Jingzhou Central Hospital, Jingzhou, Hubei, China; ^5^ Institution of Pathology, Tongji Hospital Affiliated to Tongji Medical College of Huazhong University of Science & Technology, Wuhan, Hubei, China

**Keywords:** immune checkpoint inhibitors, advanced gastric cancer, propensity score matching, progression free survival, overall survival

## Abstract

**Background:**

Immune-checkpoint inhibitors (ICIs) combined with chemotherapy have been successfully used in clinical trials to treat advanced gastric cancer. However, the efficacy and safety of first-line immunotherapy combined with chemotherapy in Chinese patients are unknown.

**Methods:**

This multicenter retrospective study included patients with human epidermal growth factor receptor-2 (HER-2) negative advanced gastric cancer treated with first-line chemotherapy or chemotherapy with an ICI between January 2019 and December 2022. Propensity score matching was used to compare progression-free survival (PFS), overall survival, objective response rates, and adverse reactions between cohorts.

**Results:**

After propensity score matching, 138 patients, who had balanced baseline characteristics, were included in the chemotherapy and combination treatment groups. The median follow-up duration was 16.90 months, and the median PFS was 8.53 months (95% confidence interval [CI] 7.77-9.28) in the combination treatment group and 5.97 months (95% CI 4.56-7.37) in the chemotherapy group. The median survival duration was 17.05 months (95% CI 14.18-19.92) in the combination treatment group and 16.46 months (95% CI 12.99-19.93) in the chemotherapy group. The PFS subgroup analysis revealed that age ≥65 years, women, Eastern Cooperative Oncology Group performance status of 1, non-signet ring cell carcinoma, esophagogastric junction, liver metastasis, peritoneal metastasis, no massive ascites, only one metastatic organ, and combined platinum-based chemotherapy correlated with treatment benefit. The incidences of adverse events above grade 3 were comparable between groups.

**Conclusions:**

Our study confirmed the ATTRACTION-4 trial results. Compared with chemotherapy, first-line ICIs combined with chemotherapy prolonged PFS but did not improve overall survival in patients with HER-2-negative advanced gastric cancer.

## 1 Introduction

Gastric cancer is a notable global health problem and the third leading cause of mortality and the sixth leading cause of morbidity (1). In China, approximately 679,000 new cases of gastric cancer and 498,000 deaths occurred in 2015, with gastric cancer ranking second in the mortality rate among malignant tumors (2). Currently, treatment methods for advanced gastric cancer are limited, and comprehensive treatment based on chemotherapy is the main strategy for advanced gastric cancer. The recommended chemotherapeutic agents for advanced gastric cancer include platinum, fluorouracil, and taxane drugs, as well as anti- human epidermal growth factor receptor-2 (HER-2) or anti-angiogenic drugs in specific populations. At present, the treatment outcome of advanced gastric cancer is unsatisfactory, and the median survival time is approximately only 1 year (3).

Recently, several clinical studies have revealed the survival benefits of immune- checkpoint inhibitor (ICI) therapy in select populations with gastric cancer. Compared with chemotherapy alone, combined immunotherapy can increase the overall response rate (ORR) while prolonging progression free survival (PFS) and overall survival (OS) in specific populations (4–7). Multiple guidelines, including those of the National Comprehensive Cancer Network, European Society for Medical Oncology, and Chinese Society of Clinical Oncology, recommend the first-line use of ICIs in combination with chemotherapy in patients with advanced gastric cancer with a high combined positive score (CPS) (3, 8, 9).

Currently, ICIs are highly accessible and widely used for the treatment of advanced gastric cancer in China. Here, we analyzed the short- and long-term outcomes and adverse reactions of patients with advanced gastric cancer treated with chemotherapy or chemotherapy combined with ICIs, to explore the efficacy and safety of immunotherapy in this patient population.

## 2 Materials and methods

### 2.1 Study design and participants

This retrospective, multicenter study involved patients diagnosed with HER-2 negative local advanced or metastatic gastric adenocarcinoma. The protocol of this study was reviewed and approved by the ethics committee of Tongji Hospital of Huazhong University of Science and Technology (Ethical approval no: TJ-IRB 20230303). All patients were fully informed about the objectives of the study, and the requirement for informed consent was waived due to this study’s observational retrospective design. This study retrospectively analyzed the clinical data of patients with advanced gastric cancer from six cancer centers across China, between January 2019 and December 2022. Data were collected from the first chemotherapy session until patient death.

All eligible patients had histologically or cytologically confirmed unresectable, locally advanced, relapsed, or metastatic gastric adenocarcinoma; had received at least one cycle of doublet or triplet chemotherapy or doublet or triplet chemotherapy combined with immunotherapy; and had been evaluated for efficacy at least once. Patients with recurrent gastric cancer were included when at least six months had elapsed from the end of adjuvant or neoadjuvant therapy. Patients were excluded if their clinical data were incomplete, survival follow-up data were not available, their HER-2 status was positive, or if they received single-agent chemotherapy.

**Figure 1:**
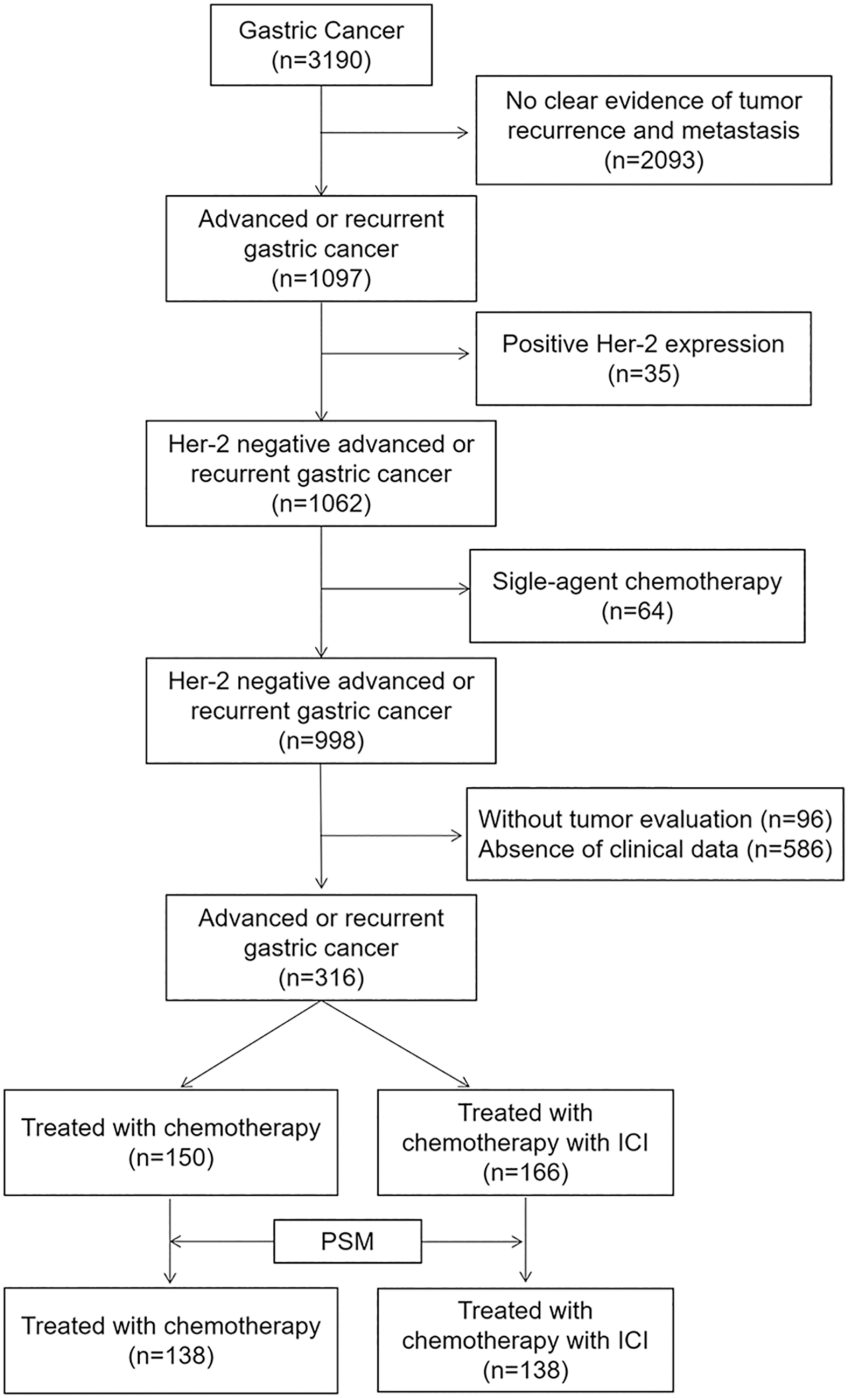
Flow diagram of the study.

After screening 3190 patients according to the above criteria, we excluded 2093 patients with no clear evidence of tumor recurrence and metastasis, 35 patients with positive HER-2 expression, 64 patients who received single-agent chemotherapy, 96 patients without tumor evaluation, and 586 patients with no readily accessible clinical data. In the final analysis, 316 patients were included (**Figure 1**).

### 2.2 Study procedures

All patients included in the final analysis received first-line oxaliplatin- or taxane-based chemotherapy and some patients received a treatment combined with ICIs, at the discretion of the clinician.

The following baseline characteristics were collected for each patient: age, sex, Eastern Cooperative Oncology Group (ECOG) performance status (PS), primary tumor location, involved organs, CPS, microsatellite instability status, and chemotherapy regimen, if available. Computed tomography scans were conducted every 6-8 weeks after the initiation of first-line chemotherapy, to evaluate the clinical response using the Response Evaluation Criteria in Solid Tumors (RECIST) version 1.1 (10).

### 2.3 Outcomes

The primary endpoint was first-line PFS, which was estimated from treatment initiation to progression or death. The secondary endpoints included OS, defined as the duration from treatment initiation to death due to any reason; ORR, defined as the number of patients with a best overall response of complete response or partial response (PR); and disease control rate (DCR), defined as the proportion of patients who achieved a complete response, PR, stable disease, or non-PR/non-Progression Disease and safety. Adverse events (AEs) were monitored and classified according to the Common Terminology Criteria for Adverse Events version 5.0.

### 2.4 Statistical analysis

Continuous and categorical data were reported as medians (interquartile range [IQR]) and percentages. PFS and OS were estimated using Kaplan-Meier analysis and expressed as median values with corresponding two-sided 95% confidence intervals (CIs), and differences between treatment groups were compared by log-rank tests with two-sided significance levels of p=0.05. The ORR and DCR were compared using the chi-square test. Hazard ratios (HRs) and corresponding 95% CIs were calculated using the Cox proportional hazards model. Univariate and multivariate analyses were performed to evaluate the effects of immunotherapy on PFS and OS.

A 1:1 propensity score matching (PSM) algorithm with a caliper of 0.1 was conducted to adjust for the non-random design of the study. The propensity score was estimated by multivariate logistic regression, with combined immunotherapy as the dependent variable, and age, sex, ECOG PS, primary tumor location, liver metastasis, signet-ring cell status, peritoneum metastasis, massive ascites, first-line chemotherapy regimen, and number of organs involved as covariables.

All statistical analyses were performed using SPSS (version 27.0, IBM Corp., Armonk, NY) and GraphPad Prism (version 9.0; GraphPad Software, San Diego, CA).

## 3 Results

### 3.1 Patient characteristics

The median age of the included patients was 55 years (IQR 48-63); 180 (57.0%) of the 316 patients were men, and all patients had an ECOG PS of 0-1. The patients received a median of five cycles (IQR 4-6) of first-line fluoropyrimidine-based (5-fluorouracil, capecitabine, S-1, etc.) chemotherapy. The majority (65.5%) of patients received platinum drug regimens (oxaliplatin, cisplatin, etc.) and 41.5% received taxane drug regimens (docetaxel, paclitaxel, nap-paclitaxel, etc.), among whom 7% received platinum combined with taxane regimens (DCF, DOX, FLOT, etc.) A total of 166 patients (52.2%) received first-line chemotherapy combined with ICIs, including nivolumab, sintilimab, tislelizumab, camrelizumab, and pembrolizumab. Because CPS values were not available for more than 90% of the enrolled patients, no analysis was performed for this indicator. Patients who received up to eight cycles of first-line treatment without disease progression and with tolerable adverse event profiles were treated with maintenance therapy, which consisted of single-agent chemotherapy (S-1 or capecitabine) combined with or without immunotherapy.

### 3.2 PSM results

After performing PSM using the procedures described in the Methods section, 138 patients who received first-line chemotherapy alone and 138 matched patients who received first-line chemotherapy combined with immunotherapy were included in the final analysis. The baseline characteristics of patients before matching revealed statistically significant differences between the groups in terms of age and proportion of first-line platinum-based chemotherapy regimens. However, the post-matching analysis revealed well-balanced characteristics between the two groups (**Table 1**).

**Table 1:** Demographic and clinical characteristics of patients before and after PSM.

	Before PSM			After PSM		
Variable	CT (n=150)	CT+ICI (n=166)	*p* value	CT (n=138)	CT+ICI (n=138)	*p* value
Sex			0.136			0.543
Male	92(61.3%)	88(53.0%)		81(58.7%)	76(55.1%)	
Female	58(38.7%)	78(47.0%)		57(41.3%)	62(44.9%)	
Age	56.5(51.0-64.0)	53.0(44.8-63.0)	0.011	55.0(49.8-63.0)	54.0(47.5-64.0)	0.395
ECOG PS			0.813			0.185
0	73(48.7%)	83(50.0%)		73(52.9%)	62(44.9%)	
1	77(51.3%)	83(50.0%)		65(47.1%)	76(55.1%)	
Primary tumor location						
EGJ	29(19.3%)	18(10.8%)		24(17.4%)	14(10.1%)	0.204
GC	119(79.3%)	145(87.3%)		112(81.2%)	121(87.7%)	
residue	2(1.3%)	3(1.8%)		2(1.4%)	3(2.2%)	
Signet-ring cell			0.413			0.457
Yes	27(18.0%)	36(21.7)		26(18.8%)	31(22.5%)	
No	123(82.5)	130(78.3%)		112(81.2%)	107(77.5%)	
Metastatic site						
Liver	42(28.0%)	44(26.5%)	0.766	37(26.8%)	36(26.1%)	0.891
Peritoneum	82(54.7%)	80(48.2%)	0.250	73(52.9%)	71(50.7%)	0.718
Number of organs involved						
1	49(32.7%)	63(38.0%)	0.327	45(32.6%)	52(37.7%)	0.377
≥2	101(67.3%)	103(62.0%)		93(67.4%)	86(62.3%)	
Massive ascites			0.312			0.651
Yes	32(21.3%)	28(16.9%)		29(21.0%)	26(18.8%)	
No	118(78.7%)	138(83.1%)		109(79.0%)	112(81.2%)	
First-line chemotherapy regimen						
Taxane-based	68(45.3%)	63(38.0%)	0.184	59(42.8%)	54(39.1%)	0.541
Platinum-based	87(58.0%)	120(72.3%)	0.008	84(60.9%)	92(66.7%)	0.316

PSM, propensity score matching; CT, chemotherapy; ICI, immune-checkpoint inhibitors; ECOG PS, Eastern Cooperative Oncology Group performance status; GC, gastric cancer; EGJ, esophagogastric junction.

### 3.3 Efficacy

**Figure 2:**
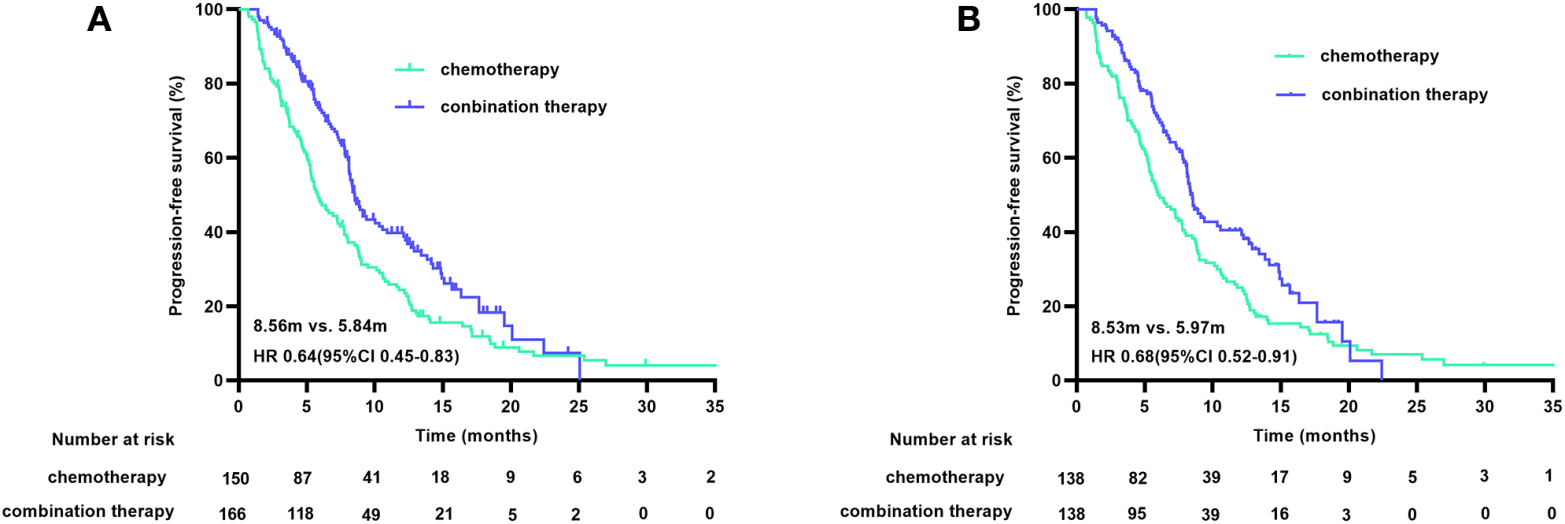
PFS curves before **(A)** and after **(B)** PSM. PFS, progression free survival; PSM, propensity score matching.

At the cutoff date of January 9, 2023, 236 of 316 patients (74.7%) had disease progression, and 207 of the 276 matched patients (75.0%) had PFS. After a median follow-up duration of 16.90 months, the median PFS (mPFS) durations before matching were 5.84 months (95% CI 4.69-6.98) in the chemotherapy group and 8.56 months (95% CI 7.86-9.26) in the combination treatment group (HR 0.64 [95% CI 0.45-0.83], p<0.001). The post-match analysis revealed that the mPFS duration in the chemotherapy group was 5.97 months (95% CI 4.56-7.37), and that in the combination treatment cohort was 8.53 months (95% CI 7.77-9.28) (HR 0.68 [95% CI 0.52-0.91], p=0.008). The PFS curves before and after matching are shown in **Figure 2**. The 6-month PFS rate was 46.4% (95% CI 38-55) with chemotherapy and 58.7% (95% CI 50-67) with combined therapy.

**Figure 3:**
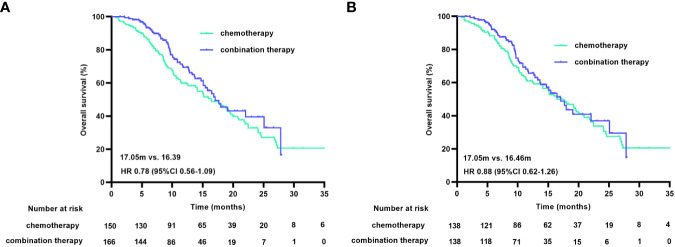
OS curves before **(A)** and after **(B)** PSM. OS, overall survival; PSM, propensity score matching.

At the cutoff date, 150 of the 316 patients had died, with a median OS (mOS) duration of 16.39 months (95% CI 12.90-19.89) in the chemotherapy group and 17.05 months (95% CI 14.12-19.97) in the combination treatment group (HR 0.78 [95% CI 0.56-1.09], p=0.147). The matched data analysis showed that 135 (48.9%) of the 276 patients had died, with mOS durations of 16.46 months (95% CI 12.99-19.93) in the chemotherapy group and 17.05 months (95% CI 14.18-19.92) in the combination treatment group (HR 0.88 [95% CI 0.62-1.26], p=0.481), with no statistical difference in OS between the two groups, either before or after matching (**Figure 3**).

In the matched population, according to RECIST1.1 criteria, one patient in the chemotherapy group achieved complete response, 41 patients (29.7%) achieved PR, and the ORR was 30.4%. In the combined treatment group, 51 patients achieved PR, no patients achieved complete response, and the ORR was 37.0%. There was no statistically significant difference in ORR between the two groups (p=0.252). The DCR in the chemotherapy group was 84.8%, which was significantly lower than that in the combination treatment group (93.5%) (p=0.020). **Supplementary Table 1** shows the tumor responses during first-line treatment in each study cohort.

### 3.4 Subgroup analysis

**Figure 4:**
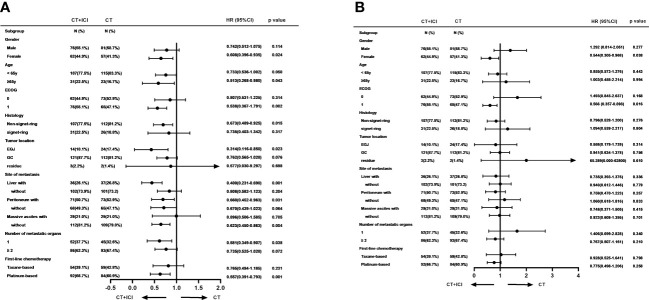
Subgroup analyses of PFS **(A)** and OS **(B)** based on baseline characteristics. PFS, progression free survival; OS, overall survival.

In the *post-hoc* subgroup analysis of PFS based on baseline characteristics, women aged 65 years or older, ECOG PS of 1, non-signet ring cell carcinoma, esophagogastric junction, liver metastasis, peritoneal metastasis, no massive ascites, and only one metastatic organ were associated with benefits from combination therapy. Moreover, in the choice of chemotherapy regimen, immunotherapy combined with a platinum-based chemotherapy regimen appeared to provide more PFS benefits. The results of the subgroup analysis of PFS and OS are shown in **Figure 4**.

This study included 59 patients without measurable target lesions who presented with peritoneal metastases or ascites. In the cohort with measurable target lesions(n=217), chemotherapy combined with immunotherapy improved the DCR by 13.5% compared to chemotherapy alone (93.7% versus 80.2%; p=0.003; **Supplementary Table 2**), while there was no significant difference in ORR (45.9% versus 39.6%; p=0.347) between the treatments. In the cohorts with no measurable target lesions, there was no difference in DCR (92.6% versus 100.0%, p=0.398) between treatments. The survival analysis showed no significant differences in PFS (7.77 m *vs*. 8.72 m, HR 0.75 [95% CI 0.53-1.04], p=0.081) and OS (15.93 m *vs*. 20.75 m, HR 0.80 [95% CI 0.54-1.21], p=0.297) between patients with or without target lesions. The corresponding PFS and OS results are shown in Online **Supplementary Figures 1, 2**.

### 3.5 Expansion follow-up

**Figure 5:**
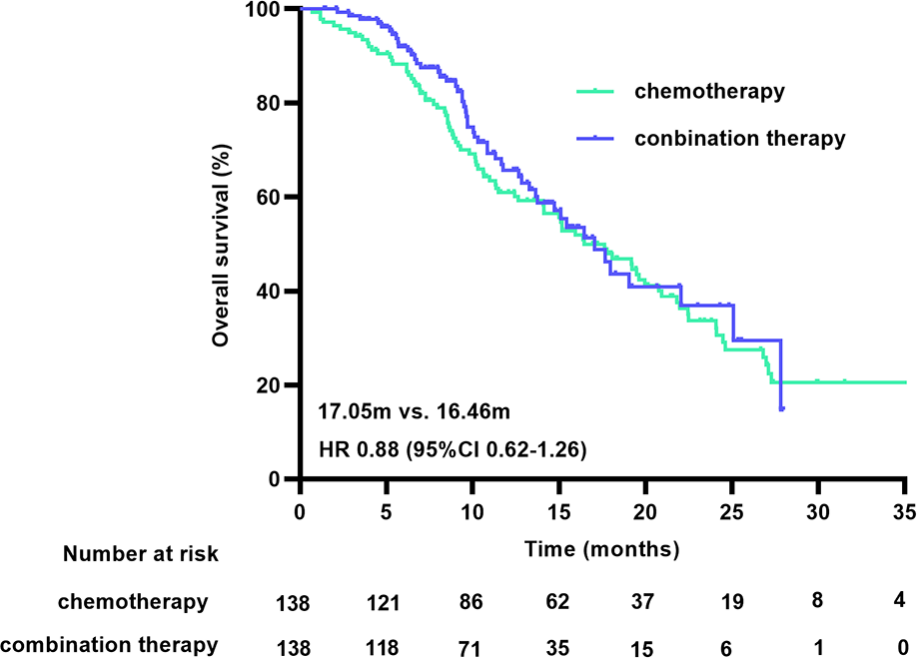
OS curve for the treatment with or without ICI. OS, overall survival; ICI, immune checkpoint inhibitors.

Of the matched patients, 77 (55.8%) of 138 patients receiving chemotherapy, and 57 (41.3%) of 138 patients receiving combination therapy received at least one subsequent anticancer therapy following progression after first-line treatment. Of the patients in the chemotherapy group, 59.7% (46/77) received immunotherapy after first-line treatment progression, and 70.2% (40/57) of the patients in the combination treatment group received continued immunotherapy after disease progression. The subgroup analysis showed that in the first-line chemotherapy group, combined immunotherapy after disease progression reduced the risk of death by 53.6%, compared with chemotherapy (HR 0.46 [95% CI 0.26-0.84], p=0.010), with an associated mOS of 24.07 months and 14.07 months, respectively. In the first-line combination treatment group, sequential immunotherapy beyond progression had no significant impact on OS (17.97 m *vs*. 13.28 m, HR 0.80 [95% CI 0.36-1.79], p=0.590) (**Supplementary Figures 3, 4**). Patients who had been treated with ICIs during first-line or sequential treatment had a significantly longer OS durations compared with patients who had never been treated with ICI [19.97 m *vs*. 11.34 m, HR 0.60 (95% CI 0.43-0.84, p=0.003)]. The associated survival curves are shown in **Figure 5**.

Of the 276 patients, 59 (21.4%) received palliative radiotherapy at various treatment stages. The corresponding treatments included radiotherapy for primary foci, liver metastases, metastatic lymph nodes, or metastatic bone lesions. The survival analysis revealed that compared to patients who did not receive palliative radiotherapy, patients who underwent palliative radiotherapy had significantly longer survival, with respective median OS durations of 21.80 months *vs*. 15.12 months (HR, 0.55 [95% CI 0.35-0.87], p=0.010). The corresponding survival curves for palliative radiotherapy are shown in **Supplementary Figure 5**.

### 3.6 Safety

The main AEs identified after matching are presented in **Table 2**. In the chemotherapy group, 92.8% (128/138) of the patients experienced some grade of AE, as did 98.6% (136/138) of patients in the combination treatment group. The most common AEs included anemia, leukopenia, neutropenia, elevated alanine aminotransferase or aspartate aminotransferase, and thrombocytopenia, most of which were of grade 1-2 and manageable.

**Table 2:** Summary of adverse events.

	CT (N=138)				CT+ICI (N=138)				*p*	
	Any		≥3 Grade		Any		≥3 Grade		Any	≥3 Grade
Hematological										
Leucopenia	74	53.6	20	14.5	82	59.4	15	10.9	0.331	0.366
Neutropenia	72	52.2	34	24.6	81	58.7	23	16.7	0.276	0.102
Anemia	118	85.5	24	17.4	116	84.1	18	13.0	0.738	0.315
Thrombocytopenia	49	35.5	8	5.8	66	47.8	6	4.3	0.038	0.583
Non-hematological										
ALT/AST increase	49	35.5	1	0.7	76	55.1	2	1.4	0.001	0.583
Creatinine increase	9	6.5	0	0	8	5.8	1	0.7	0.830	1.000
Total bilirubin increase	14	10.1	1	0.7	13	9.4	0	0	0.839	0.316
Albumin decrease	36	26.1	1	0.7	48	34.8	0	0	0.116	0.316
hypothyroidism	NA	NA	NA	NA	18	13.0	0	0	NA	NA
Hyperthyroidism	NA	NA	NA	NA	4	2.9	0	0	NA	NA
Hypophysitis	NA	NA	NA	NA	2	1.4	0	0	NA	NA
Amylase/lipase evaluation	NA	NA	NA	NA	7	5.1	0	0	NA	NA
myocarditis	NA	NA	NA	NA	2	1.4	1	0.7	NA	NA
pneumonitis	NA	NA	NA	NA	2	1.4	0	0	NA	NA

CT, chemotherapy; ICI, immune checkpoint inhibitors; ALT/AST, alanine aminotransferase/aspartate aminotransferase; NA, not available.

Any grade thrombocytopenia and elevated alanine aminotransferase or aspartate aminotransferase were significantly more frequent in the combination treatment group; however, there was no difference in the incidence of AEs above grade 2 between the two cohorts. ICI-related thyroid dysfunction occurred in 15.9% of the patients, most of whom had hypothyroidism. Overall, hyperthyroidism occurred in 2.9% of the patients, all of whom eventually developed hypothyroidism. Two patients developed myocarditis, one of whom developed cardiogenic shock, and ICI therapy was discontinued in both patients. Acute renal failure occurred in one patient; however, it was difficult to determine whether the adverse reaction was an immune-related AE. The remaining immune-related AEs were grade 1-2.

## 4 Discussion

In this multicenter, retrospective, real-world study, chemotherapy combined with ICI therapy was found to significantly improve PFS in previously untreated HER-2 negative patients with advanced gastric adenocarcinoma. This regimen reduced the risk of disease progression by 32% compared with chemotherapy. Consistent with the results of previous clinical studies, our results reveal that ICIs combined with chemotherapy provide clinical benefits to patients with advanced HER-2 negative gastric adenocarcinoma (4–7).

However, our results showed no significant difference in OS between the groups, which is inconsistent with the results of some previous clinical studies. In the CheckMate 649 and Orient 16 studies, chemotherapy combined with immunotherapy was associated with a significant improvement in OS in all randomly assigned patients and was more pronounced in patients with high CPS expression. In our study, 64.7% of the patients received sequential treatment after disease progression following first-line treatment, which was similar to the incidence reported in the ATTRACTION-4 study, whereas the incidence was only 39% in the CM649 study. It is widely accepted that patients who received subsequent anticancer pharmacotherapy had better survival. More than 60% of the patients who received sequential therapy chose combination immunotherapy, and patients who received immunotherapy throughout the course of their treatment had a 40% lower risk of death than those who did not. This finding suggests that the use of immunotherapy as a sequential therapy may provide survival benefits, even if first-line immunotherapy is not used. This finding can be explained by the fact that patients in the first-line chemotherapy group, chemotherapy combined with immunotherapy after disease progression significantly prolongs OS and increases HR benefits.

In the KEYNOTE-062 study, pembrolizumab plus chemotherapy was not superior to chemotherapy alone in terms of OS and PFS; however, immunotherapy combined with chemotherapy has been associated with significant improvements in both parameters in multiple studies, including CheckMate 649, ATTRACTION-4, and Orient 16 (11). Cisplatin-based chemotherapy was used in the KEYNOTE-062 study, whereas oxaliplatin-based chemotherapy was used in other clinical studies. Programmed cell death protein 1 (PD-1) inhibitors combined with oxaliplatin-based chemotherapy may be a better first-line treatment option for patients with advanced gastric cancer (12). In our study, 60.9% of the matched patients in the combination therapy group received platinum-based chemotherapy, but only four patients received a cisplatin-containing regimen, with the remainder receiving oxaliplatin, and 36.2% of the patients were treated with taxane-based chemotherapy. Preclinical studies have shown that paclitaxel can activate antitumor immunity by inducing immunogenic cell death, which increases PD-L1 expression within the tumor microenvironment, stimulates natural killer cells and T lymphocytes, and affects macrophage polarization, thereby enhancing PD-1 antibody efficacy (13–21). This combination may be more effective than other chemotherapeutic agents, such as cisplatin and oxaliplatin (13).

A previous clinical study conducted by our research team showed that first-line chemotherapy with albumin-paclitaxel plus S-1 resulted in prolonged PFS in patients with HER-2-negative advanced gastric cancer, compared with first-line chemotherapy with oxaliplatin plus S-1 (22). However, the results of the survival analysis in this study showed that when combined with immunotherapy, patients who received taxanes as first-line chemotherapy had slightly longer PFS and OS than those who received platinum-based drugs, although the differences were not statistically significant. Interestingly, the results of the PFS subgroup analysis suggested that patients benefited more from the addition of immunotherapy when platinum-based chemotherapy was selected as first-line treatment. The effects of chemotherapeutic agents on the immune microenvironment are complex and subtle, and further studies are needed to confirm which chemotherapeutic agents are the best combinations for immunotherapy.

Previous randomized clinical trial results have revealed that patients with liver metastases, ECOG PS of 1, and non-signet ring cell carcinoma were more likely to benefit from immunotherapy, which is consistent with the results of this study (5–7). In the subgroup analysis of the RATIONAL305 study, immunotherapy combined with chemotherapy significantly prolonged OS in patients without peritoneal metastasis, while no significant survival benefit was shown in patients with peritoneal metastasis (7). Our study included 59 patients without target lesions who presented with peritoneal metastases or massive ascites, a population that was excluded from prospective randomized controlled trials but represents a substantial proportion of patients in the real world. Our results showed no difference in PFS and OS between patients with and without target lesions, and the subgroup analysis results suggested that immunotherapy could impact survival benefit to patients with peritoneal metastasis but could not impact obvious benefits to patients with massive ascites. However, owing to the small sample size of only 59 patients, the accuracy of this result needs to be verified in a larger, diverse patient population. Notably, this population, which was excluded from prospective studies, is worthy of specific attention, and further studies are needed to investigate the efficacy of immunotherapy. Previous studies have confirmed that palliative radiotherapy plays an important role in relieving bleeding, obstruction, and pain and in improving the quality of life of patients with advanced gastric cancer; however, the relationship between palliative radiotherapy and survival is unclear (23–25). A total of 59 patients with advanced gastric cancer who received palliative radiotherapy during the course of the disease were included in this study, and the corresponding results showed that palliative radiotherapy improved their OS. Due to the small sample size, further subgroup analyses were not performed to explore whether radiotherapy could increase the efficacy of immunotherapy; however, it is well known that radiotherapy may increase the benefits of immunotherapy (26, 27). Whether palliative radiotherapy combined with chemotherapy and immunotherapy can provide survival benefits for patients with advanced gastric cancer warrants further investigation.

As this was a retrospective real-world study, clinical data collection was based on the extraction of electronic medical records and patient follow-up. Data for the safety analysis mainly came from medical records and objective laboratory and imaging examinations. Data on subjective AEs, such as rash, diarrhea, and peripheral neurotoxicity were partly missing; therefore, these subjective AEs were not included in the final safety analysis. Cardiotoxicity occurred in 1.4% of the patients in our study, which were consistent with the results of the previous studies (28, 29). One patient developed cardiogenic shock with a marked elevation in cardiac troponin levels, which resolved after treatment with high-dose corticosteroids. Overall, although chemotherapy combined with immunotherapy was associated with a low incidence of grade 3 or higher AEs and was generally well tolerated, patients with serious immune-related AEs, including cardiac and renal injuries, should be closely monitored.

A major limitation of this study was the absence of PD-L1 CPS expression results in most patients. Owing to the obvious heterogeneity of CPS detection, many pathology centers, including ours, do not perform routine CPS detection, which results in a large amount of missing data (30). Although high PD-L1 expression has been confirmed to be a good independent prognostic factor for survival in previous clinical studies, CPS was not further analyzed in this study due to missing data (31, 32). In this study, four patients had deficient mismatch repair/microsatellite instability-high tumors, and only one patient achieved PFS. Therefore, the relationship between the mismatch repair status and survival was not analyzed. Despite the use of PSM, the potential biases caused by the retrospective, non-randomized design remains a limitation of this study.

## 5 Conclusion

The findings of this PSM study showed that first-line treatment with chemotherapy combined with ICIs significantly improved PFS in patients with HER-2 negative advanced gastric cancer; however, there was no significant improvement in OS, and the side effects were tolerable. The results of this study are consistent with those of ATTRACTION-4 and confirm the efficacy and safety of immunotherapy in combination with chemotherapy in a real-world setting.

## Data availability statement

The original contributions presented in the study are included in the article/**Supplementary Material**. Further inquiries can be directed to the corresponding authors.

## Ethics statement

The studies involving humans were approved by the ethics committee of Tongji Hospital of Huazhong University of Science and Technology. The studies were conducted in accordance with the local legislation and institutional requirements. The ethics committee/institutional review board waived the requirement of written informed consent for participation from the participants or the participants’ legal guardians/next of kin because the requirement for informed consent was waived due to this study’s observational retrospective design.

## Author contributions

YD: Data curation, Investigation, Software, Writing – original draft. YL: Data curation, Writing – original draft. ZG: Resources, Writing – original draft. LH: Resources, Writing – original draft. LW: Data curation, Writing – original draft. WY: Data curation, Writing – original draft. PQ: Resources, Writing – original draft. FZ: Data curation, Writing – original draft. XY: Conceptualization, Methodology, Writing – review & editing. HC: Conceptualization, Methodology, Writing – review & editing. HQ: Conceptualization, Methodology, Writing – review & editing.
