# Supplementary figures and images for: Revalidation of the ATTRACTION-4 study in a real-world setting: a multicenter, retrospective propensity score matching study in China
Source: Front Immunol. 2023 Sep 15;14:1264929. doi: 10.3389/fimmu.2023.1264929 (PMC10541969; doi:10.3389/fimmu.2023.1264929)

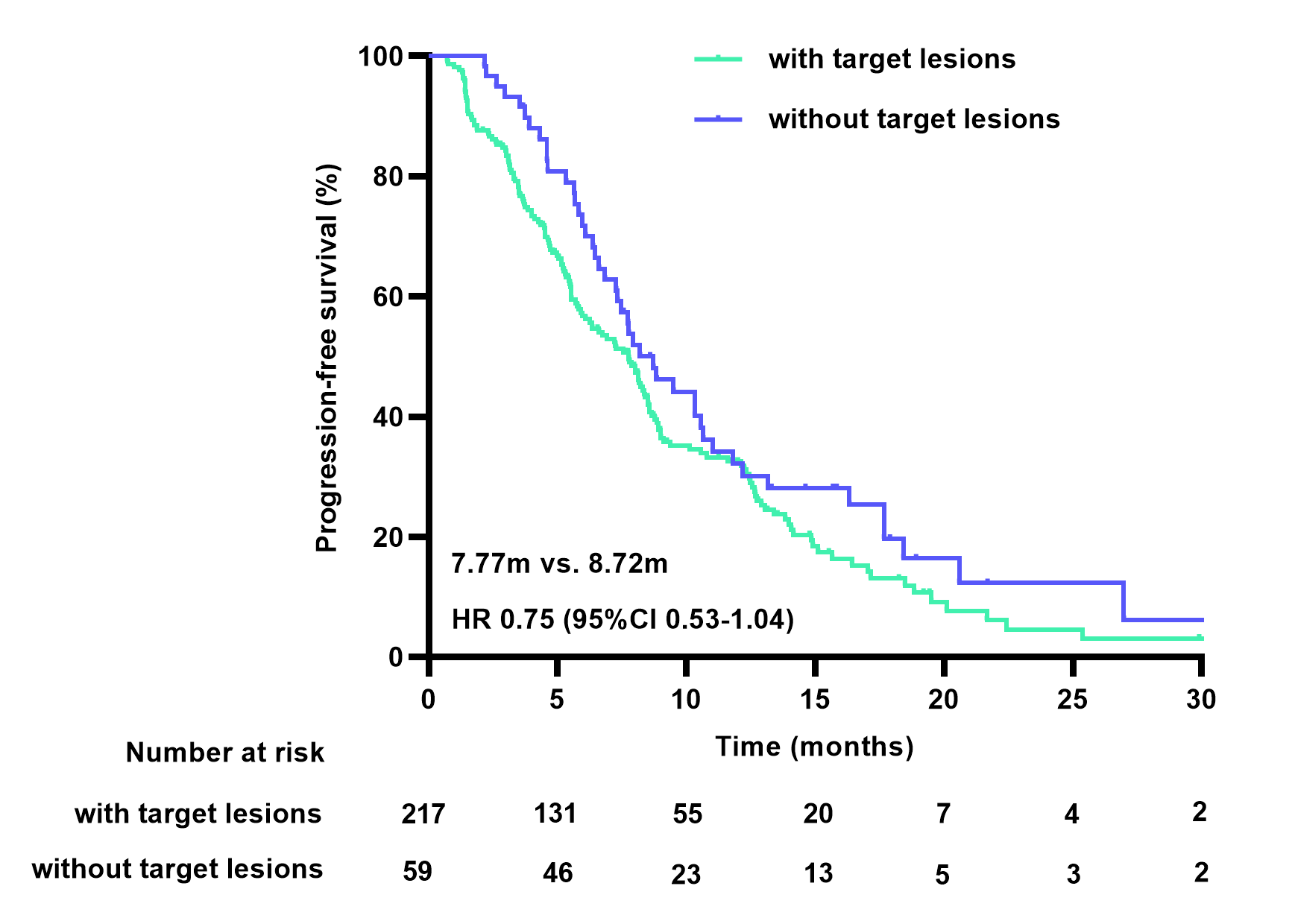

Supplement: Supplementary file 1 [file Image_1.tif]

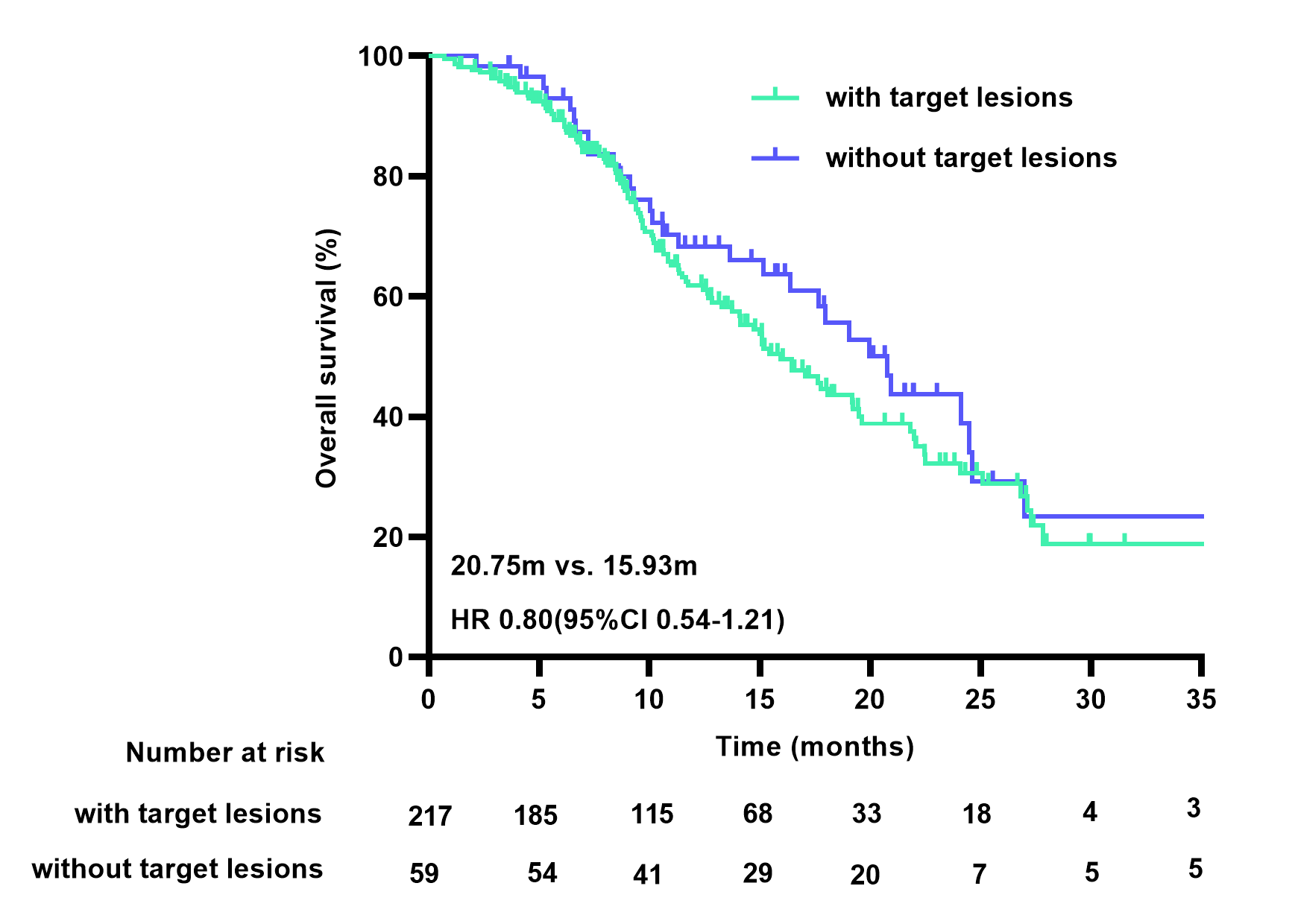

Supplement: Supplementary file 2 [file Image_2.tif]

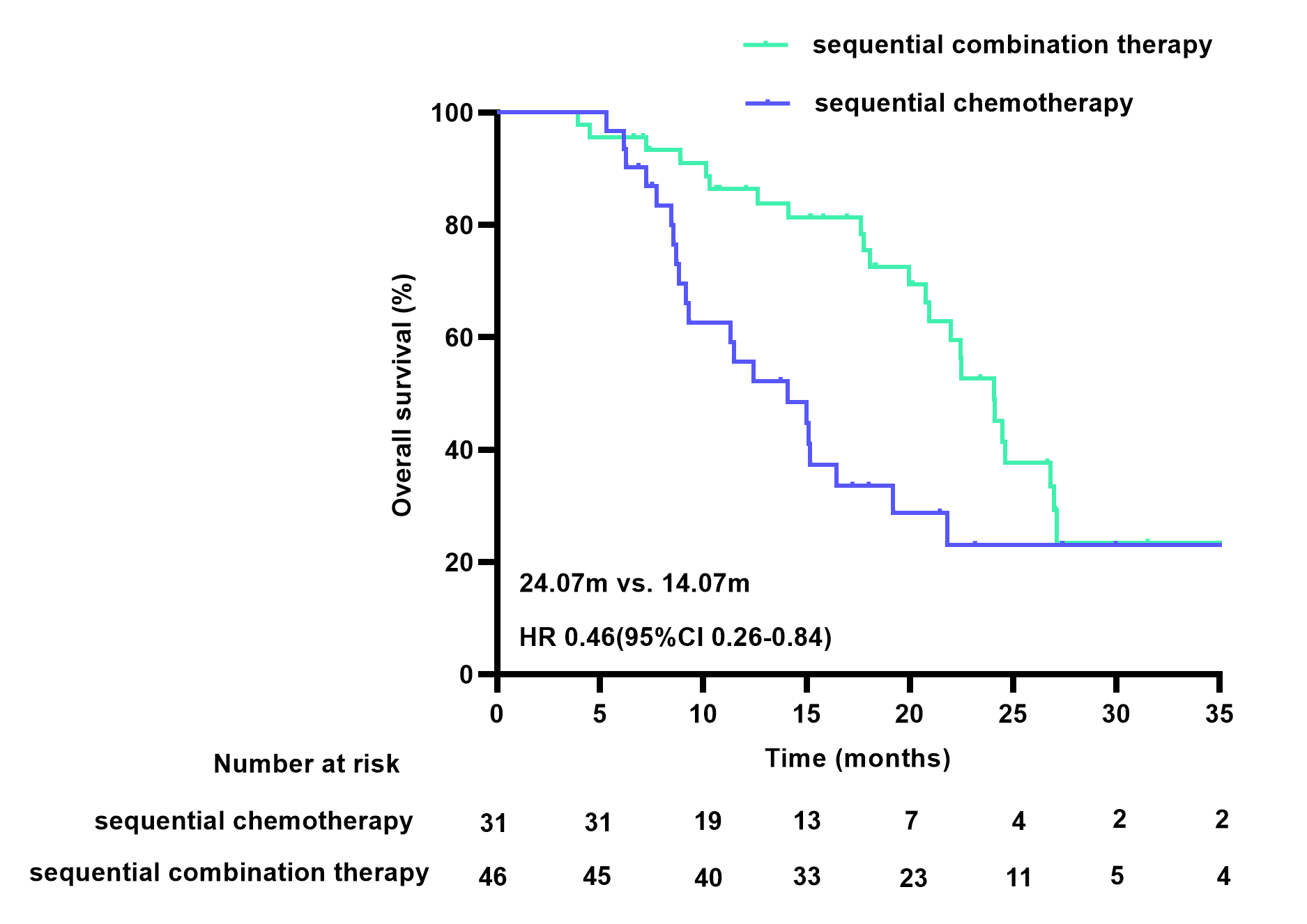

Supplement: Supplementary file 3 [file Image_3.tif]

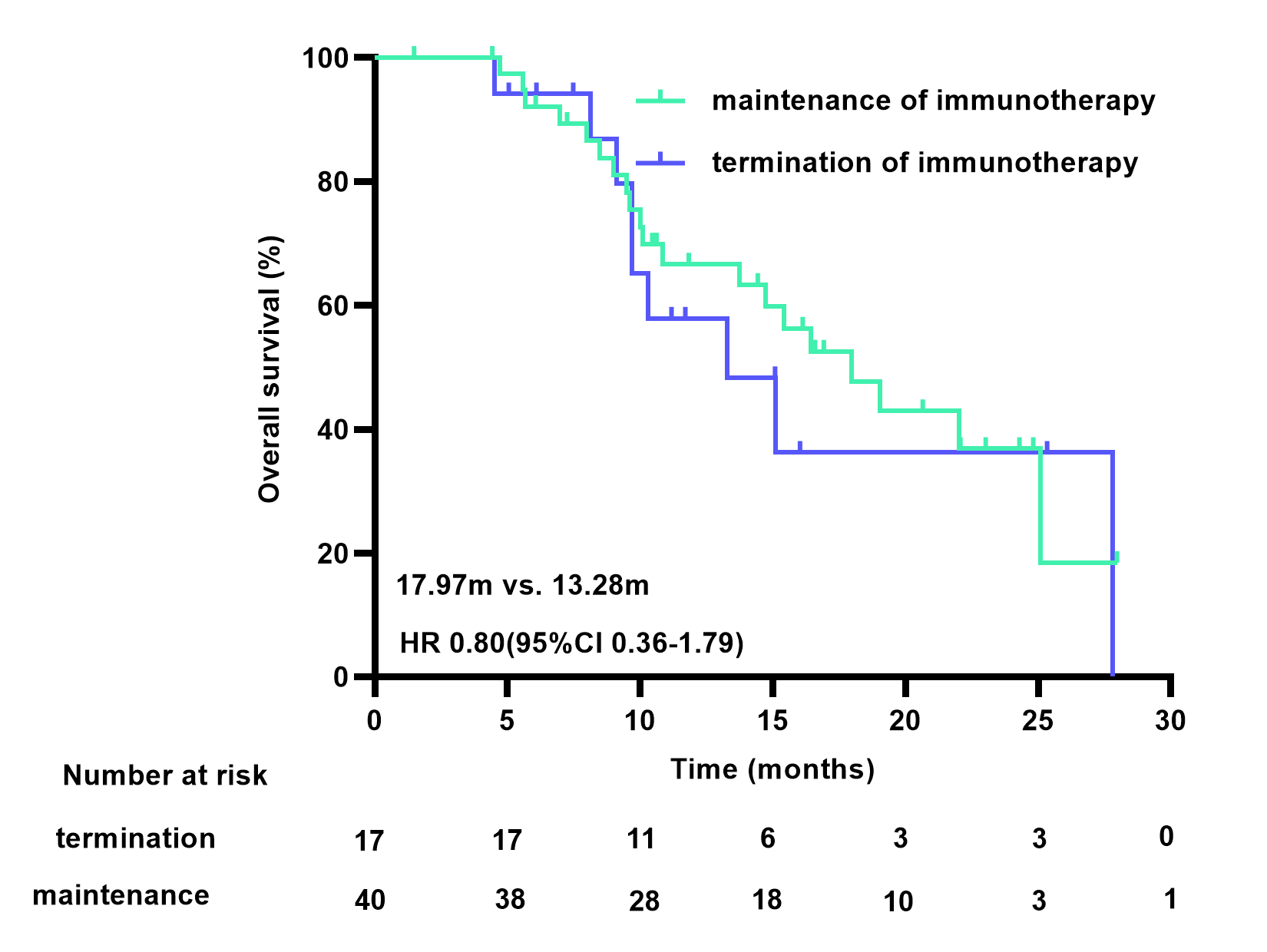

Supplement: Supplementary file 4 [file Image_4.tif]

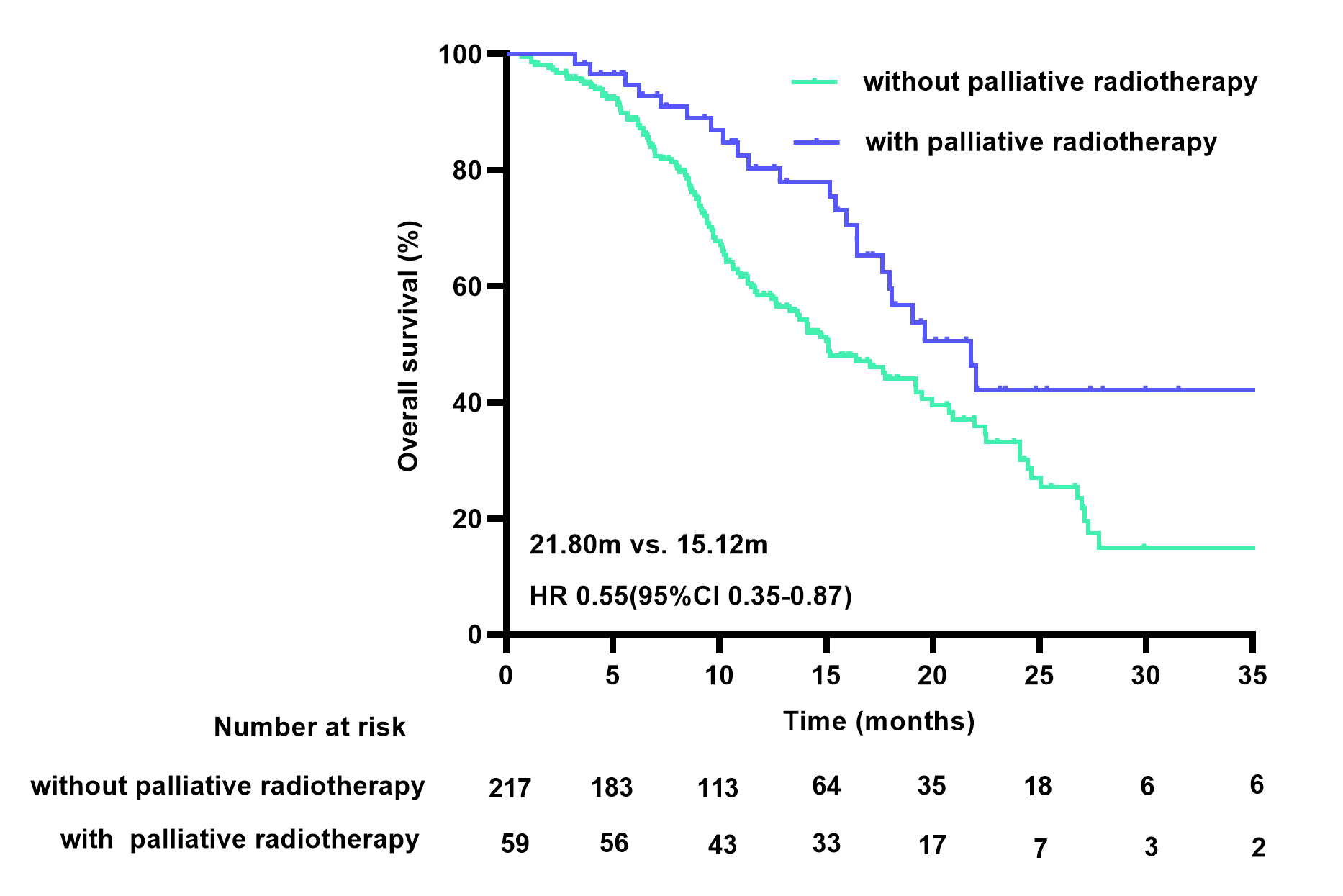

Supplement: Supplementary file 5 [file Image_5.tif]
