# Supplementary material for: Revalidation of the ATTRACTION-4 study in a real-world setting: a multicenter, retrospective propensity score matching study in China
Source: Front Immunol. 2023 Sep 15;14:1264929. doi: 10.3389/fimmu.2023.1264929 (PMC10541969; doi:10.3389/fimmu.2023.1264929)
Supplement: Supplementary file 6 [file Table_1.docx]

Supplementary Table 1 Tumor response according to RECIST 1.1

|  | CT  (n=138) | CT+ICI  (n=138) | *p* value |
| --- | --- | --- | --- |
| CR | 1 (0.7%) | 0 (0) |  |
| Partial response | 41 (29.7%) | 51 (37.0%) |  |
| Stable disease | 43 (31.2%) | 53 (38.4%) |  |
| Progressive disease | 21 (15.2%) | 9 (6.5%) |  |
| Non-CR/non-PD | 32 (23.2%) | 25 (18.1%) |  |
| Objective response rate (95%CI) | 30.4% (22.7%-38.2%) | 37.0% (28.8%-45.1%) | 0.252 |
| Disease control rate (95%CI) | 84.8% (78.7%-90.9%) | 93.5% (89.3%-97.6%) | 0.020 |

RECIST, Response Evaluation Criteria in Solid Tumors; CT, chemotherapy; ICI, immune checkpoint inhibitors; CR, complete response; PD, progression disease; CI, confidence interval
