# Supplementary material for: Revalidation of the ATTRACTION-4 study in a real-world setting: a multicenter, retrospective propensity score matching study in China
Source: Front Immunol. 2023 Sep 15;14:1264929. doi: 10.3389/fimmu.2023.1264929 (PMC10541969; doi:10.3389/fimmu.2023.1264929)
Supplement: Supplementary file 7 [file Table_2.docx]

Supplementary Table 2 Subgroup analysis of tumor response according to measurable disease (RECIST 1.1)

|  | Measurable disease (n=217) | | | Non-measurable disease (n=59) | | |
| --- | --- | --- | --- | --- | --- | --- |
|  | CT (n=106) | CT+ICI (n=111) | p value | CT (n=32) | CT+ICI (n=27) | p value |
| Complete response | 1 (0.9) | 0 (0%) | 0.592 | 0 (0.00) | 0 (0.00) |  |
| Partial response | 41(38.7%) | 51 (45.9%) |  | 0 (0.00) | 0 (0.00) |  |
| Stable disease | 43 (40.6%) | 53 (47.7%) |  | 0 (0.00) | 0 (0.00) |  |
| Progressive disease | 21(19.8%) | 7 (6.3%) |  | 0 (0) | 2 (7.4%) |  |
| non-CR/non-PD | 0 (0.00) | 0 (0.00) |  | 32 (100%) | 25 (92.6%) |  |
| Objective response (95%CI) | 42 (39.6%; 30.2-49.1) | 51(45.9%; 36.5-55.4) | 0.347 | 0 (0.00) | 0 (0.00) | - |
| Disease control (95%CI) | 85 (80.2%; 72.5-87.9) | 104(93.7%; 89.1-98.3) | 0.003 | 32 (100%; 100.0-100.0) | 25 (92.6%; 82.0-103.2) | 0.398 |

RECIST, Response Evaluation Criteria in Solid Tumors; CT, chemotherapy; ICI, immune checkpoint inhibitors; CR, complete response; PD, progression disease; CI, confidence interval
